# Ketogenic diet alleviates colitis by reduction of colonic group 3 innate lymphoid cells through altering gut microbiome

**DOI:** 10.1038/s41392-021-00549-9

**Published:** 2021-04-23

**Authors:** Cheng Kong, Xuebing Yan, Yongqiang Liu, Linsheng Huang, Yefei Zhu, Jide He, Renyuan Gao, Matthew F. Kalady, Ajay Goel, Huanlong Qin, Yanlei Ma

**Affiliations:** 1 Department of Gastrointestinal Surgery, Shanghai Tenth People’s Hospital, Tongji University, Shanghai, China; 2 Research Institute of Intestinal Diseases, Tongji University School of Medicine, Shanghai, China; 3 Department of Cancer Biology, Lerner Research Institute, Cleveland Clinic, Cleveland, OH USA; 4 Department of Oncology, Affiliated Hospital of Yangzhou University, Yangzhou University, Yangzhou, China; 5 Department of Colorectal Surgery, Digestive Disease and Surgery Institute, Cleveland Clinic, Cleveland, OH USA; 6 Department of Molecular Diagnostics and Experimental Therapeutics, Beckman Research Institute of City of Hope Comprehensive Cancer Center, Duarte, CA USA; 7 Department of Oncology, Shanghai Medical College, Fudan University, Shanghai, China; 8 Department of Colorectal Surgery, Fudan University Shanghai Cancer Center, Shanghai, China

**Keywords:** Gastrointestinal diseases, Gastrointestinal diseases

## Abstract

Accumulating evidence suggests that ketogenic diets (KDs) mediate the rise of circulating ketone bodies and exert a potential anti-inflammatory effect; however, the consequences of this unique diet on colitis remain unknown. We performed a series of systematic studies using a dextran sulfate sodium (DSS) animal model of inflammatory colitis. Animals were fed with a KD, low-carbohydrate diet (LCD), or normal diet (ND). Germ-free mice were utilized in validation experiments. Colon tissues were analyzed by transcriptome sequencing, RT2 profiler PCR array, histopathology, and immunofluorescence. Serum samples were analyzed by metabolic assay kit. Fecal samples were analyzed by 16S rRNA gene sequencing, liquid chromatography–mass spectrometry and gas chromatography–mass spectrometry. We observed that KD alleviated colitis by altering the gut microbiota and metabolites in a manner distinct from LCD. Quantitative diet experiments confirmed the unique impact of KD relative to LCD with a reproducible increase in *Akkermansia*, whereas the opposite was observed for *Escherichia/Shigella*. After colitis induction, the KD protected intestinal barrier function, and reduced the production of RORγt^+^CD3^−^ group 3 innate lymphoid cells (ILC3s) and related inflammatory cytokines (IL-17α, IL-18, IL-22, Ccl4). Finally, fecal microbiota transplantation into germ-free mice revealed that the KD- mediated colitis inhibition and ILC3 regulation were dependent on the modification of gut microbiota. Taken together, our study presents a global view of microbiome-metabolomics changes that occur during KD colitis treatment, and identifies the regulation of gut microbiome and ILC3s as novel targets involving in IBD dietary therapy.

## Introduction

Inflammatory bowel disease (IBD) is a complicated chronic inflammatory disease that involves various genetic and environmental driving factors.^1^ Although its pathogenesis is poorly understood, an increasing number of studies have highlighted that dietary intake plays a key role in disease occurrence due to its underlying effects on gut microbiota, barrier function, and mucosal immunity.^2^ For instance, a high-salt diet has been shown to exacerbate intestinal inflammation by reducing *Lactobacillus* abundance and butyrate metabolism,^3^ while a high-fat diet has been found to contribute toward IBD progression by activating proinflammatory signaling and disrupting barrier systems.^4^ Although exclusive enteral nutrition has shown clinical promise for promoting Crohn’s disease remission, it remains unclear whether dietary therapy would help to treat ulcerative colitis.^5^ Therefore, the discovery of novel anti-inflammatory dietary strategies is urgently required to manage IBD.

A ketogenic diet (KD), characterized by high-fat and low-carbohydrate, is a dietary therapy that was initially used to treat drug-resistant epilepsy,^6^ but has recently gained increasing popularity as a promising therapeutic approach for chronic diseases and human malignancies. In Alzheimer’s disease, a KD not only protects aging nerve cells but also enhances mitochondrial function and downregulates the expression of apoptotic mediators.^7^ A KD has also been shown to contribute toward reducing diet-induced glycemic responses and thus may represent a favorable adjuvant therapy for type 2 diabetes mellitus.^8^ In addition, a strict KD was found to alter amino acid metabolism in cancer cells, inhibit oncogenic epigenetic modifications, and enhance the efficacy of chemo- and radio-therapies.^9^ A recent review attributed the therapeutic potential of KD to its regulation of gut microbiota and related metabolites, yet emphasized that a KD may also reduce bacterial diversity.^10^ A newly published work revealed KD-associated gut microbiota decrease the accumulation of intestinal Th17 cells, implying its potential anti-inflammatory role.^11^ Despite emerging evidences proposing its clinical application in numerous diseases, few studies have investigated whether a KD could be used to alleviate intestinal inflammation or examined its effect on gut microbiota and immune cells in inflammatory microenvironments.

In this study, we compared the gut microbiome and metabolic changes in mice fed with a KD, low-carbohydrate diet (LCD), or normal diet (ND). In addition, we investigated the effect of a KD on inflammation, the gut microbiome, mucosal barriers, and immunity using a dextran sulfate sodium (DSS)-induced colitis model. Finally, we clarified the beneficial effects of the KD-modified gut microbiome on intestinal inflammation using fecal microbiota transplantation (FMT) in a germ-free (GF) mouse model. Our findings suggest that KD is a promising dietary therapy for intestinal inflammation and demonstrate its modulatory effect on gut microbiota.

## Results

### KD contributes to beneficial gut microbiota and related metabolites in mice before colitis induction

**Fig. 1:**
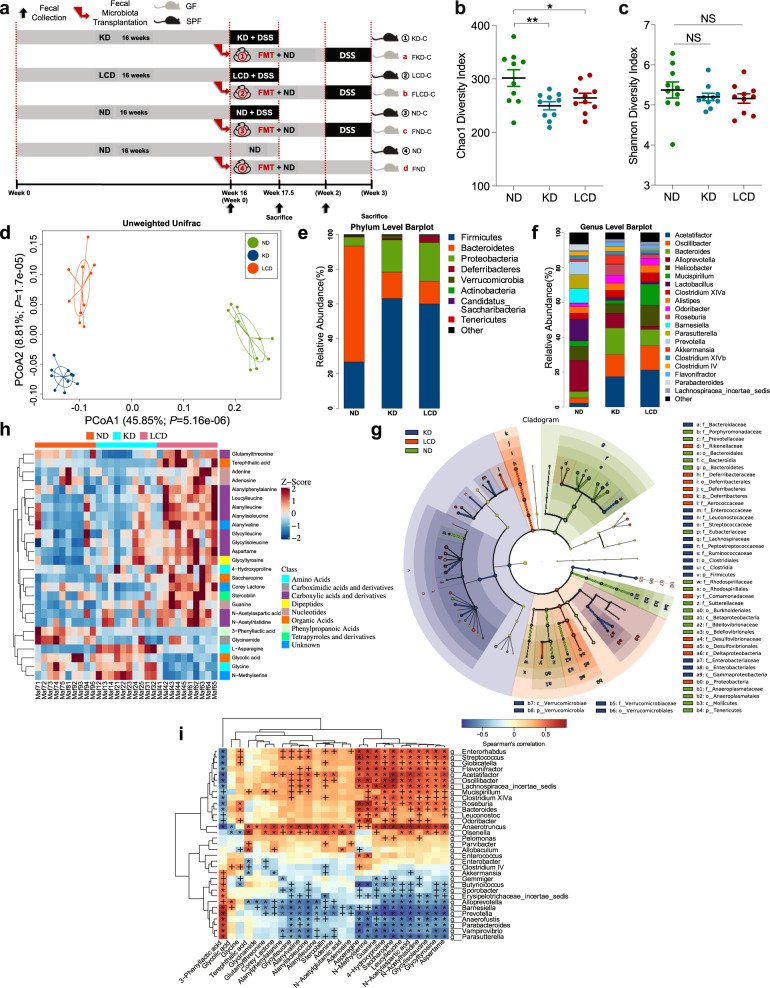
KD contributes to beneficial gut microbiota and related metabolites in mice before colitis induction. **a** Schematic representation of study design. DSS-induced C57BL/6J colitis mice were fed with a KD, LCD, or ND and sacrificed at 17.5 weeks. DSS-induced C57BL/6J colitis germ-free mice received FMT from diet-treated mice and were sacrificed at 19 weeks. **b**, **c** α-diversity Chao1 (**b**) and Shannon indices (**c**) of mice fed with a KD, LCD, or ND for 16 weeks (*p < 0.05; **p < 0.01). **d** PCoA scores based on the relative abundance of operational taxonomic units (97 % similarity). Each symbol represents a sample. **e**, **f** Dominant phyla (**e**) and genera (**f**) in each group. **g** Cladogram of linear discriminant analysis scores for differentially abundant bacteria (p: Phylum, c: Class, o: Order, f: Family). **h** Clustering analysis of different metabolites in each group. **i** Correlation heatmap of gut microbiota and metabolites (^+^*p* < 0.05; *p < 0.01). (*n* = 10 per group). Results are expressed as mean ± SEM (**b**, **c**). KD, ketogenic diet; LCD, low-carbohydrate diet; ND, normal diet; DSS, dextran sulfate sodium; FMT, fecal microbiota transplantation; PCoA, principal component analysis

Before inducing colitis, we analyzed the effects of a KD and LCD on the fecal microbiota of mice after 16 weeks of dietary intervention (Fig. 1a). As shown in Fig. 1b, the Chao1 index was significantly lower in the KD and LCD groups than in the ND group; however, no significant differences in the Shannon index were observed between the KD/LCD and ND groups (Fig. 1c). Unweighted PCoA analysis indicated a clear distinction between the KD, LCD, and ND groups (Fig. 1d). At the phylum level (Fig. 1e), the Firmicutes/Bacteroidetes ratio and relative abundance of Proteobacteria were higher in the KD and LCD groups than in the ND group. At the genus level (Fig. 1f), *Akkermansia* and *Roseburia* were more abundant in the KD group than in the other groups, while *Alloprevotella* is most abundant in ND. Linear discriminant analysis coupled with effect size measurements (LEfSe) revealed that *Oscillibacter* was enriched in the LCD group while *Akkermansia*, *Roseburia*, and *Ruminococcaceae* were enriched in the KD group (Fig. 1g, Supplementary Fig. 1a). The functional potential of gut microbiota was analyzed using PICRUSt and LEfSe (Linear Discriminant Analysis (LDA) score > 3.0, *p* < 0.05), indicating that bacterial motility proteins and bacterial chemotaxis were over-represented in the microbiome of the LCD group compared to the KD and ND groups, while the function “bacterial invasion of epithelial cells” was over-represented in the KD group (Supplementary Fig. 1b).

To clarify the effects of a KD and LCD on bacterial metabolites, we used GC-MS and UHPLC-HRMS/MS to identify the fecal metabolite spectra of mice after 16-weeks dietary intervention. Partial least squares-discriminant analysis (PLS-DA) score plots showed a distinct separation between the metabolites of the three groups (Supplementary Fig. 1c), of which 26 were screened as being significantly different and divided into two clusters based on their concentration profiles, including metabolite clusters dominant in LCD (e.g., terephthalic acid, stercobilin) and KD/ND (e.g., l-asparagine, glycolic acid; Fig. 1h). Details of these metabolites are provided in Supplementary Table 1. Straightforward associations between bacteria and metabolites were demonstrated using correlation heatmaps (Fig. 1i), indicating that KD-dominated *Akkermansia* and ND-dominated *Alloprevotella* correlated negatively with several LCD-dominant metabolites such as terephthalic acid and stercobilin. These results suggest that quantitative induction of ketogenesis relative to ND can effectively alter the gut microbiota and metabolites.

### KD controls the body weight gain and changes the metabolic status of mice

**Fig. 2:**
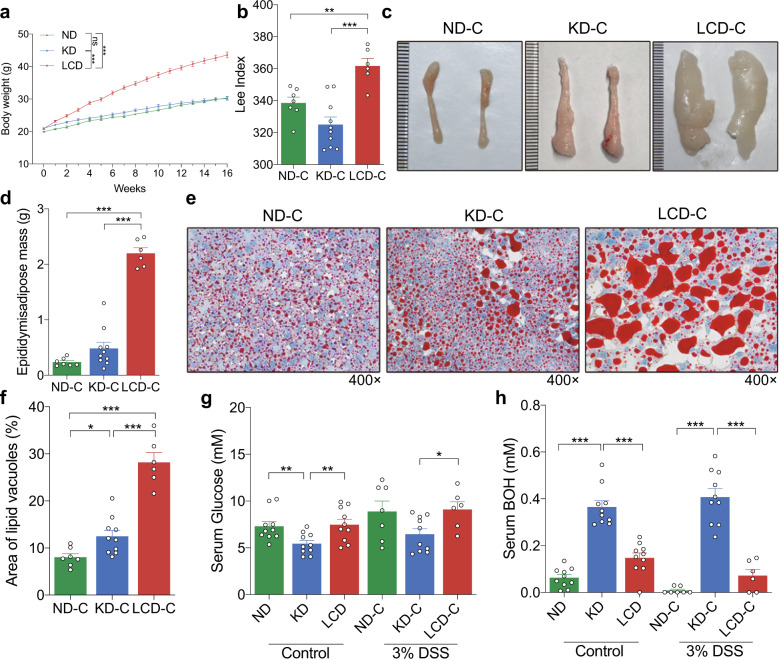
KD controls the body weight gain and changes the metabolic status of mice. **a** Body weight of mice fed with a KD, LCD, or ND for 16 weeks before colitis induction. **b** Lee’s index comparison among KD-C, LCD-C and ND-C groups. **c**, **d** Representative images of epididymal white adipose tissues and tissue weight comparison of ND-C, KD-C and LCD-C groups. **e**, **f** Representative liver oil red O staining images and liver lipid accumulation. Original magnification ×400. **g** Serum glucose levels in mice before and after DSS-induced colitis. **h** Serum BOH levels in mice before and after DSS-induced colitis. *p < 0.05; **p < 0.01; ***p < 0.001. (*n* = 10 for KD, LCD, ND, and KD-C; LCD-C, *n* = 6; ND-C, *n* = 7). Results are expressed as mean ± SEM (**a**, **b**, **d, f–h**). KD, ketogenic diet; LCD, low-carbohydrate diet; ND, normal diet; DSS, dextran sulfate sodium; BOH, 3-hydroxybutyric acid

As shown in Fig. 2a, LCD significantly promoted weight gain in mice before colitis induction whereas no significant weight gain difference was observed between the KD and ND groups, consistent with the Lee index of colitis-induced mice (Fig. 2b). We also measured the weight of fat in the epididymal white adipose tissues of colitis-induced mice, finding that it was significantly higher in the LCD-C group than in the KD-C or ND-C groups (Fig. 2c, d). Similar results were observed when comparing the fat vesicles of livers from each group (Fig. 2e, f). Finally, we detected that serum glucose levels were significantly lower in the KD group than in the LCD or ND groups (Fig. 2g), whereas serum BOH levels were significantly higher (Fig. 2h). These results demonstrated, LCD significantly promoted the body weight, while KD significantly altered the glucose and BOH metabolism of the mice before and after colitis induction.

### KD alleviates inflammation and protects mucosal barrier function in mice with DSS-induced colitis

**Fig. 3:**
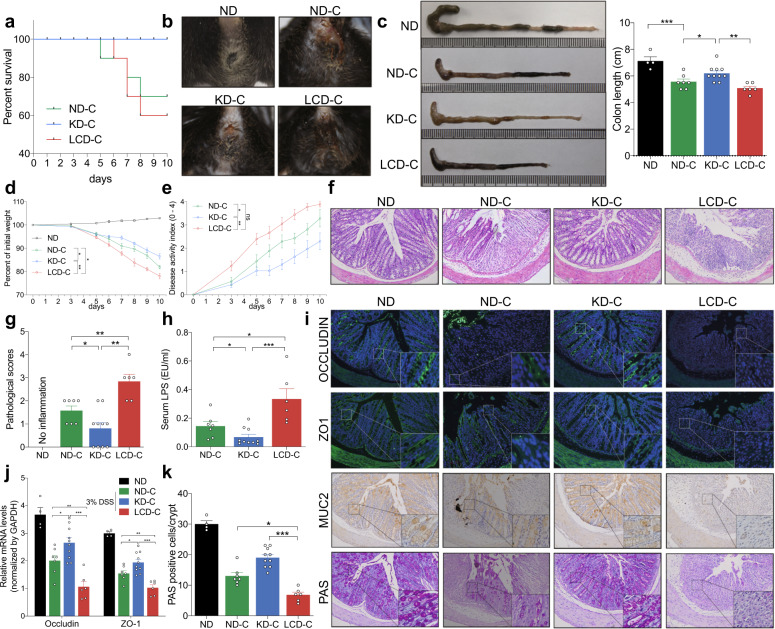
KD alleviates inflammatory responses and protects mucosal barrier function in mice with colitis. **a** Survival curves of mice with DSS-induced colitis fed with a KD, LCD, or ND. **b** Representative images of bloody diarrhea. **c** Representative images of the colon at 1.5 weeks after DSS induction. Colon length shown as a chart. **d** Body weight change in mice receiving 3% DSS in drinking water. **e** DAI in each group. **f** Representative colon H&E staining images (original ×magnification 200). **g** Changes in colonic section pathological scores. **h** Serum LPS levels detected by end-point chromogenic tachypleus amebocyte lysate. **i** Representative images of immunofluorescence, IHC and PAS staining in colon tissues with antibodies against Occludin, ZO-1, and MUC-2 (original magnification ×200). **j** Colon Occludin and ZO-1 mRNA expression measured by qRT-PCR. **k** Number of PAS-positive cells per villus/ crypt. *p < 0.05; **p < 0.01; ***p < 0.001. (KD-C, *n* = 10; LCD-C, *n* = 6; ND-C, *n* = 7; ND, *n* = 4). Results are expressed as mean ± SEM (**c–e**, **g**, **h**, **j**, **k**). KD ketogenic diet, LCD low-carbohydrate diet, ND normal diet, DSS dextran sulfate sodium, LPS lipopolysaccharide, IHC immunohistochemistry, PAS periodic acid schiff

The survival curves of each group following induction of colitis are shown in Fig. 3a. None of the mice in the KD-C group died during the experiment, whereas four and three mice died in the LCD-C and ND-C groups, respectively. In addition, mice in the LCD-C group were more likely to have hemafecia and shortened colons than those in the KD-C or ND-C groups, while mice in the KD-C group had less hemafecia and prolonged colons than in the ND-C group (Fig. 3b, c). Furthermore, mice in the KD-C group had less weight loss and disease activity index (DAI) scores than those in the ND-C group, while the opposite was observed in the LCD-C group (Fig. 3d, e). H&E staining revealed that the KD-C group displayed less inflammatory cell infiltration in the intestinal epithelium than the ND-C group, whereas the LCD-C group displayed more infiltration (Fig. 3f, g). Finally, the KD lowered serum LPS levels compared to the ND, but the LCD increased LPS levels (Fig. 3h).

Next, we evaluated the effect of KD and LCD on barrier function in mice with DSS-induced colitis by using immunofluorescence and immunohistochemistry to detect barrier-related proteins in harvested colon tissues (Fig. 3i). The KD significantly increased Occludin, ZO-1, and MUC-2 expression in the intestinal epithelium compared to the ND, whereas the opposite was observed for the LCD. These findings were confirmed by quantitative PCR (Fig. 3j), while PAS staining revealed that the KD and LCD increased and decreased the number of goblet cells in the colon compared to the ND, respectively (Fig. 3k). Our results highlight how KD are distinctive from LCD in both the inflammatory colitis and intestinal barrier function.

### KD and LCD affect mucosal inflammation and immunity in mice with DSS-induced colitis

**Fig. 4:**
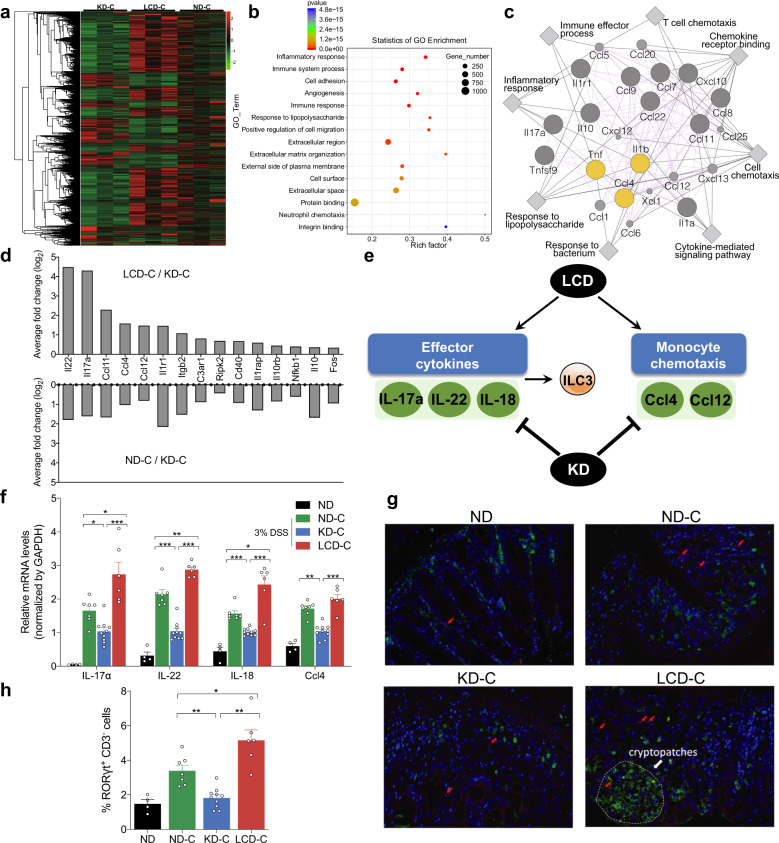
Effect of KD and LCD on mucosal inflammation and immunity in mice with colitis. **a** Heatmap of 1643 differentially expressed genes among three groups: KD-C (DSS-induced colitis mice fed with KD, *n* = 3), LCD-C (DSS-induced colitis mice fed with LCD, *n* = 3), ND-C (DSS-induced colitis mice fed with ND, *n* = 3). **b** Gene ontology (GO) functional analysis shows the top 15 biological functions that are enriched in the significantly expressed genes. **c** Cytoscape software predicts the interaction between top 20 genes selected according to their corrected *p*-values and fold change values. **d** Significant upregulated expression of 15 transcripts in LCD-C, KD-C, ND-C groups detected by mouse inflammatory response and autoimmunity PCR arrays. **e** Systematic diagram of major inflammatory pathways and immune cells potentially regulated by the 15 upregulated genes. **f** Quantitative RT-PCR confirming altered IL-17α, IL-18, IL-22, and Ccl4 expression. **g** Representative immunofluorescence images of RORγt^+^CD3^−^ ILC3 cells in colon tissues (original magnification ×400). Green: positive staining of RORγt; Red arrows: positive staining of CD3. **h** Percentage of RORγt^+^CD3^−^ ILC3 cells in colonic sections. *p < 0.05; **p < 0.01; ***p < 0.001. (KD-C, *n* = 10; LCD-C, *n* = 6; ND-C, *n* = 7; ND, *n* = 4). Results are expressed as mean ± SEM (**f, h**). KD ketogenic diet, LCD low-carbohydrate diet, ND normal diet, DSS dextran sulfate sodium, ILC3 group 3 innate lymphoid cell

To investigate the underlying mechanisms for KD or LCD-mediated colitis and barrier function changes, we performed the transcriptome sequencing using the colon tissues from KD, LCD and ND after colitis induction (*n* = 3 per group). Using a cut off of *p* < 0.01, 1643 differentially expressed genes were selected (Fig. 4a and Supplementary Table 2). To understand the biological functions potentially regulated by these genes, further investigations based on Gene Ontology (GO) database were made. The results revealed these genes may be involved in inflammatory response, immune system process, immune response, response to lipopolysaccharide, and neutrophil chemotaxis (Fig. 4b). Then, Cytoscape software was used to predict the interaction between top 20 genes selected according to their corrected *p*-values and fold change values (Fig. 4c). Among the gene sets significantly enriched in LCD-C, genes associated with cell chemotaxis (Ccl4, Ccl11, IL-1β) and immune/inflammatory response (Tnf, IL-10, IL-1r1, IL-17α) were present and interacted. To validate the transcriptome sequencing, additional mouse inflammatory response and autoimmunity PCR arrays were used to profile the expression of 84 key genes involved in inflammatory response affected by KD or LCD (*n* = 4 per group). The data showed up-regulation of 15 candidate genes in the LCD-C vs. KD-C groups (Fig. 4d). Literature review identified two mucosal inflammation- and immunity-related pathways implicated by these genes: ILC3 effector cytokines (including IL-17α, IL-22, and IL-18) and monocyte chemotaxis (including Ccl4 and Ccl12; Fig. 4e). Notably, IL-22 may directly regulate barrier function or indirectly exert this role through producing IL-18.^12^ Thus, PCR assays validated that colon tissues from the KD-C and LCD-C groups displayed lower and higher IL-22, IL-17α, IL-18, and Ccl4 expression than the ND-C group, respectively (Fig. 4f). Furthermore, immunofluorescence indicated that colon epithelium from the LCD-C and ND-C groups was enriched with more RORγt^+^CD3^−^ ILC3 cells than the KD-C group, most of which were concentrated in cryptopatches. (Fig. 4g, h). Taken together, we found that the inflammatory cytokines, chemokines and ILC3 cells are the targets of KD in inhibiting colitis.

### KD and LCD affect the gut microbiota of mice with DSS-induced colitis

**Fig. 5:**
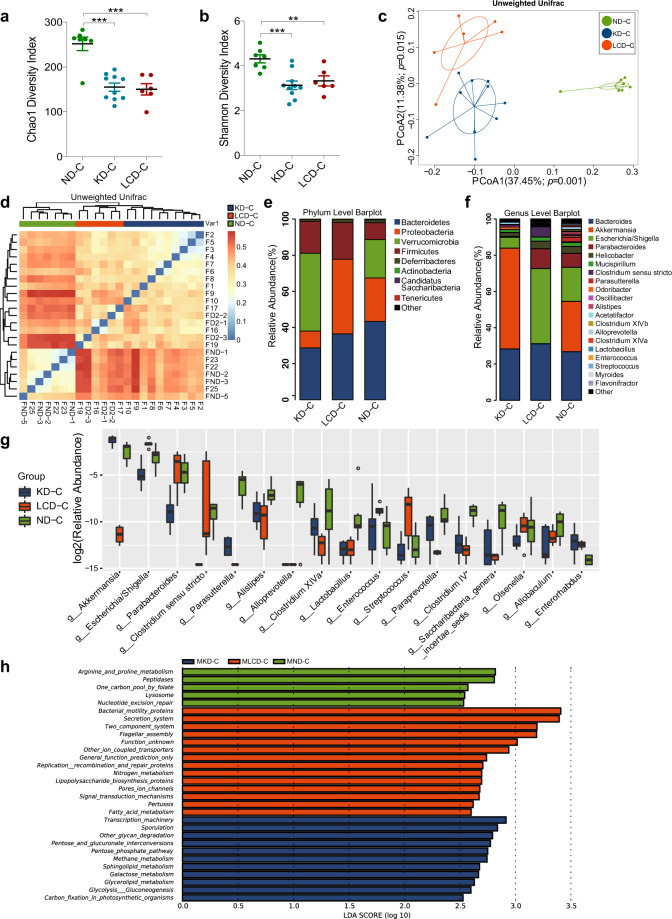
Effects of KD and LCD on gut microbiota in mice with colitis. Effects of KD and LCD on gut microbiota in mice with colitis. **a**, **b** α-diversity Chao1 (**a**) and Shannon indices (**b**) in mice with DSS-induced colitis fed with a KD (KD-C), LCD (LCD-C), or ND (ND-C). **c** PCoA scores based on the relative abundance of operational taxonomic units (97% similarity). Each symbol represents a sample. **d** Unweighted UniFrac distance heatmap for each group. **e**, **f** Dominant phyla (**e**) and genera (**f**) in each group. **g** Relative abundance of genera between groups (Wilcoxon rank-sum test). The box presented the 95% CIs; the line inside denotes the median. **h** Differentially abundant KEGG pathways in the MLN of each group identified by LEfSe. LDA score >2.5, *p* < 0.05. **p < 0.01; ***p < 0.001. (KD-C, *n* = 10; LCD-C, *n* = 6; ND-C, *n* = 7; MKD-C, *n* = 10; MLCD-C, *n* = 6; MND-C, *n* = 7). Results are expressed as mean ± SEM (**a**–**b**). KD ketogenic diet, LCD low-carbohydrate diet, ND normal diet, DSS dextran sulfate sodium, PCoA principal component analysis, MLN mesenteric lymph node, LEfS linear discriminant analysis effect size

To clarify the effect of the KD and LCD on gut microbiota in mice with colitis, we utilized 16S rDNA profiling. The KD-C and LCD-C groups had lower Chao1 and Shannon indices than the ND-C group (Fig. 5a, b), while unweighted PCoA analysis demonstrated a clear distinction between the three groups (Fig. 5c). A heatmap based on unweighted UniFrac distance demonstrated three sample clusters representing the KD-C, LCD-C, and ND-C groups, respectively (Fig. 5d). At the phylum level (Fig. 5e), there were significantly fewer Proteobacteria in the KD-C group than in the ND-C group but more in the LCD-C group, whereas the opposite pattern was observed for Verrucomicrobia. At the genus level (Fig. 5f, g), the gut microbiota of the KD-C and LCD-C groups were enriched with *Akkermansia* and *Escherichia/Shigella*, respectively. In addition, the relative abundance of *Parasutteralla*, *Alloprevotella*, *Lactobacillus* and *Clostridium IV* were decreased in both LCD-C and KD-C group as compared with ND-C group.

We also analyzed the microbiota present in the mesenteric lymph nodes (MLNs) of mice with colitis, finding that the Shannon and Simpson indices were significantly lower in the LCD-C group than in the KD-C or ND-C groups (Supplementary Fig. 2a). Consistent with our findings in the intestinal feces, LEfSe analysis of microbiota in MLNs revealed that *Akkermansia* was enriched in the KD-C group while *Escherichia/Shigella* were enriched in the LCD-C group (Supplementary Fig. 2b, c). Finally, KEGG pathway analysis suggested that the microbiota in the MLNs of the LCD-C group were involved in bacterial motility and lipopolysaccharide biosynthesis, while those in the KD-C group were involved in pentose and glucuronate interconversion, carbohydrate metabolism, glycolysis, and gluconeogenesis (Fig. 5h).

### FMT from KD- and LCD-fed mice affects inflammation development and barrier function in colitis-induced GF mice

**Fig. 6:**
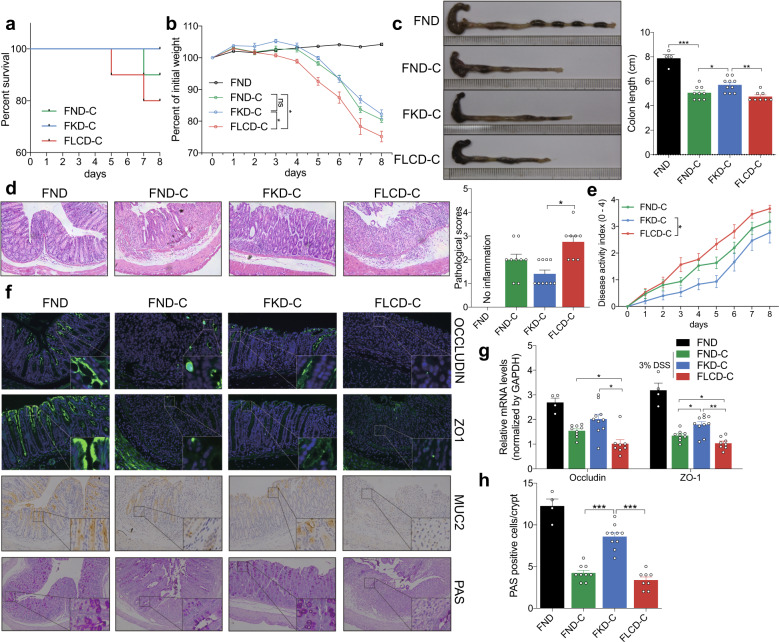
Effect of FMT from KD- and LCD-fed mice on inflammatory responses and mucosal barrier function in colitis-induced germ-free mice. **a** Survival curves of DSS-induced colitis germ-free mice after FMT from mice fed with KD (FKD-C), LCD (FLCD-C), or ND (FND-C; control). **b** Body weight change in mice receiving 3% DSS in drinking water. **c** Representative colon images on 1 week after DSS induction and colon length shown as a chart. **d** Representative colon H&E staining images (original magnification ×200). Right: colonic section pathological scores. **e** DAI in each group. **f** Representative immunofluorescence, IHC and PAS staining images of colon tissues with antibodies against Occludin, ZO-1, and MUC-2 (original magnification ×200). **g** Colon Occludin and ZO-1 mRNA expression measured by qRT-PCR. **h** Number of PAS-positive cells per villus/crypt. *p < 0.05; **p < 0.01; ***p < 0.001. (FKD-C, *n* = 10; FLCD-C, *n* = 8; FND-C, *n* = 9; FND, *n* = 4). Results are expressed as mean ± SEM (**b**–**e**, **g**, **h**). KD ketogenic diet, LCD low-carbohydrate diet, ND normal diet, DSS dextran sulfate sodium, DAI disease activity index, IHC immunohistochemistry, PAS periodic acid schiff

To validate whether the effect of KD or LCD was dependent on gut microbiota, we performed FMT from mice fed with a KD, LCD, or ND into GF mice (FKD-C, FLCD-C, and FND-C). No FKD-C mice died during the experiment; however, two died in the FLCD-C group and one died in the FND-C group (Fig. 6a). The FLCD-C group lost weight significantly faster than the FKD-C and FND-C groups (Fig. 6b), while the FKD-C group had a longer colon and lower mucosal inflammation than the FND-C group and the FLCD-C group displayed the opposite pattern (Fig. 6c, d). In addition, the FKD-C and FLCD-C groups had lower and higher DAI scores than the FND-C group, respectively (Fig. 6e). Moreover, expression of the barrier-related molecules Occludin and ZO-1 were significantly higher and lower in the FKD-C and FLCD-C groups than in the FND-C group (Fig. 6f, g). Furthermore, the FKD-C group had more goblet cells than the FND-C group, whereas the FLCD-C group had fewer (Fig. 6h).

**Fig. 7:**
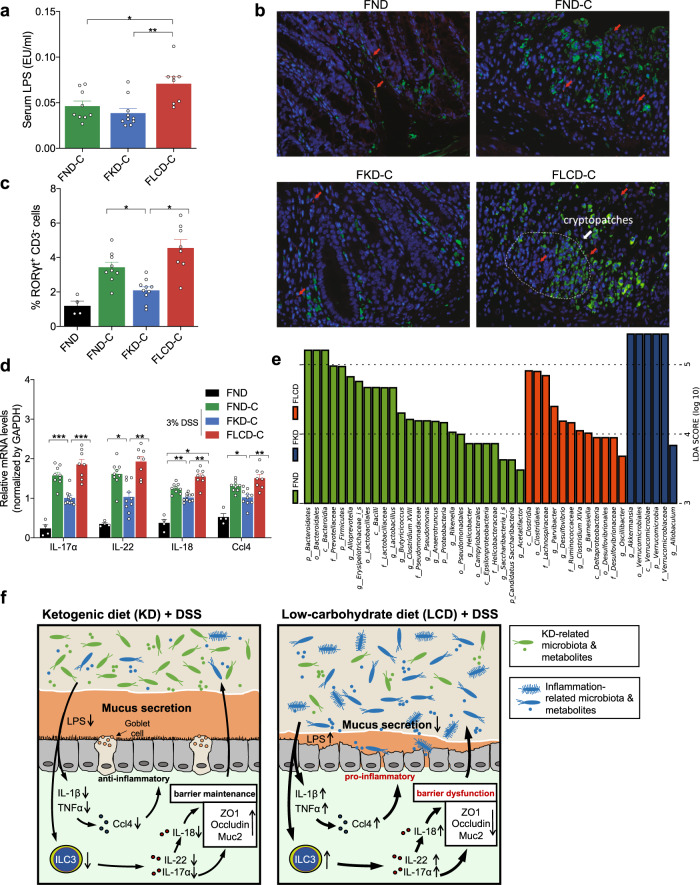
Effects of FMT from KD- and LCD-fed mice on mucosal inflammation and immunity in colitis-induced germ-free mice. **a** Serum LPS levels detected by end-point chromogenic tachypleus amebocyte lysate in DSS-induced colitis germ-free mice after FMT from mice fed with KD (FKD-C), LCD (FLCD-C), or ND (FND-C). **b** Representative immunofluorescence images of RORγt^+^CD3^−^ ILC3 cells in colon tissues (original magnification ×400). Green: positive staining of RORγt; Red arrows: positive staining of CD3. **c** Percentage of RORγt^+^CD3^−^ ILC3 cells in colonic sections. **d** Quantitative RT-PCR confirming changes in IL-17α, IL-18, IL-22, and Ccl4 expression. **e** Histogram of linear discriminant analysis scores for differentially abundant bacteria in each group (p: Phylum, c: Class, o- Order, f: Family, g: Genus). **f** Schematic summary for the proposed role of KD (left) and LCD (right) in colitis. *p < 0.05; **p < 0.01; ***p < 0.001. (FKD-C, *n* = 10, FLCD-C, *n* = 8; FND-C, *n* = 9; FND, *n* = 4). Results are expressed as mean ± SEM (**a**, **c**, **d**). KD ketogenic diet, LCD low-carbohydrate diet, ND normal diet, DSS dextran sulfate sodium, OTU operational taxonomic units, PCoA principal component analysis, ILC3 group 3 innate lymphoid cells

Next, we investigated whether FMT from diet-treated mice affected inflammatory responses or mucosal immunity. We found that serum LPS levels were lower in the FKD-C group and higher in the FLCD-C group compared to the FND-C group (Fig. 7a), while immunofluorescence indicated that the colonic epithelium of the FKD-C group was enriched with fewer RORγt^+^CD3^−^ ILC3 cells than that of the FLCD-C and FND-C groups, most of which were concentrated in cryptopatches. (Fig. 7b, c). Quantitative reverse transcription PCR (qRT-PCR) demonstrated that colon tissues from the FKD-C group displayed significantly lower IL-17α, IL-18, IL-22, and Ccl4 mRNA levels than the FND-C group, whereas the opposite was observed for the FLCD-C group (Fig. 7d). Fecal microbiota profiles confirmed significant differences between the genera present in the microbiota of all three recipient groups, including those affected by the KD (*Akkermansia*), LCD (*Oscillibacter*), and ND (*Alloprevotella* and *Lactobacillus*) that were still dominant in the FKD, FLCD, and FND groups, respectively (Fig. 7e). Detailed analysis revealed that the OTUs successfully transferred from ND microbiota accounted for 98.0% of the donor’s entire community, while those from KD and LCD microbiota accounted for 92% and 93%, respectively (Supplementary Table 3). Of the 80 most abundant OTUs in each microbiota type (88% of ND microbiota, 94% of KD microbiota, 93% of LCD microbiota), we verified that 68 KD OTUs and 72 LCD OTUs were detected in the recipient mice, whereas 78 ND OTUs were present (Fig. S3 and Supplementary Table 3). These results demonstrate that a significant proportion of OTUs successfully colonized the gut of recipient mice, validating the reliability of FMT.

## Discussion

In this study, we found that the KD and LCD changed the specific composition and function of gut microbiota and metabolites. After colitis induction, the KD significantly reduced inflammatory responses, protected intestinal barrier function, and reduced ILC3 production and the expression of related inflammatory cytokines, whereas the opposite effects were observed for the LCD. In addition, FMT revealed that the inhibitory effect of the KD in colitis was dependent on its modification of gut microbiota. Altogether, these findings highlight that KD could represent a promising dietary therapeutic strategy for treating IBD (Fig. 7f).

In this study, mice fed with a KD for 16 weeks following a quantitative feeding strategy appeared to display similar weight gain to the control group. This observation is consistent with a recent study demonstrating that a KD increases lifespan in adult mice and preserves physiological function in aged mice.^13^ We also investigated the effect of diet on gut microbiota and found that the KD dramatically increased the abundance of *Akkermansia* and butyric acid-producing *Roseburia*. Consistently, a recent study showed that a KD modulates host metabolism to prevent seizures by increasing the abundance of *Akkermansia,*^14^ while previous studies have associated several *Akkermansia* species with improved glucose homeostasis, modulating immune responses, and protecting barrier function.^15–17^ *Roseburia* is a “lean bacteria” associated with weight loss and improved glucose tolerance;^18^ therefore, the increased abundance of *Akkermansia* and *Roseburia* may be, at least in part, indirectly responsible for weight control and maintaining healthy gut physiology. The LCD mainly increased the relative abundance of *Oscillibacter*, a typical inflammation-related bacteria that is enriched in high-fat diets and is related to the increased susceptibility of progeny to colitis.^19^ The LCD critically altered microbiome functional profiles such as activated bacterial motility and LPS biosynthesis pathways, which are common in IBD animal models and accompanied by changes in intestinal barrier function.^20,21^ Over-represented bacterial invasion of epithelial cells function in KD suggests a more intensified direct interaction between bacteria and intestinal epithelial cells.

In general, our global nontargeted metabolomics analysis of fecal samples from the KD, LCD, and ND groups yielded unique and differential metabolic signatures. The feces of mice fed with a KD displayed increased levels of metabolites such as l-asparagine, glycolic acid, and glycine. Interestingly, l-asparagine exerts anti-obesity and anti-inflammatory effects in animals fed with a high-fat diet and meanwhile protects the intestinal barrier and reduces the damage caused by LPS.^22,23^ In addition, glycolic acid can act as a lipase inhibitor in the intestine to promote weight loss.^24^ Glycine is a constituent amino acid of the endogenous antioxidant that reduces glutathione and its supplementation can improve gut microbiota and exert anti-obesity effects.^25,26^ In contrast, terephthalic acid, saccharopine, stercobilin and N-acetylaspartic acid were the major metabolites in the feces of LCD-fed mice. In particular, terephthalic acid levels were ~17 times higher than in the KD-fed mice and has previously been associated with mucosal toxicity.^27^ Saccharopine (a type of mitochondrial toxin) and stecobilin have both been shown to damage cells and induce DNA-damage,^28,29^ while a recent study demonstrated that N-acetylaspartic acid can promote oxidative stress, damage antioxidant enzymes, and promote inflammation via multiple pathways, such as TNFα, iNOS, COX2, and ICAM3.^30^ Taken together, the increased levels of these metabolites in KD- and LCD-fed mice may partly explain weight change and inflammation inhibition/promotion.

To investigate the effect of the KD and LCD on intestinal barrier function and gut microbiota in inflammatory microenvironment, we used DSS to induce experimental colitis. The LCD exacerbated DSS-induced colitis and induced colonic barrier disruption, whereas the KD caused the opposite effect. The LCD significantly downregulated Occludin and ZO-1, two components of tight junctions, and Muc2, a protein secreted by goblet cells that is essential for the structure of the mucus layer in the intestine.^31^ Although many studies have reported that high-fat diets can impair intestinal barrier function, we know very little about the effect of a KD on the intestinal barrier.^32^ To explain the differences in colitis caused by the two diets, we tested gut microbiota after DSS treatment, finding that *Escherichia/Shigella* was the dominant genus in LCD-treated colitis, while KD-treated mice were dominated by *Akkermansia*. Moreover, we detected bacteria similar in composition and diversity in the MLN to those detected in stools. *Akkermansia* exerts vital inflammatory protective roles and the predominance of Proteobacteria, specifically adherent-invasive *Escherichia coli* strains in IBD, has been known for many years;^33^ however, it remains unclear whether this reflects a true driver of disease or just a change in the gut. Since both processes are likely, the diversity of disease course and presentation may represent different levels of microbial involvement in different individuals.^34^

Colitis occurrence involves changes in many genes related to immune cell trafficking, cell viability, and signal transduction.^35,36^ Surprisingly, we found that the LCD upregulated the expression of multiple chemokines and cytokines, and increased the number of ILC3 cells in the colonic lamina propria; however, the KD had the opposite effect. Chemokines are chemotactic cytokines that can promote leukocyte migration to areas of inflammation and initiate immune cell activation.^37^ The KD also reduced the expression of Ccl4, which has been associated with IBD.^38^ It has been reported that human colonic chemokine expression is non-selectively upregulated in IBD and that the degree of local inflammation and tissue damage is dependent on the local expression of specific chemokines in IBD tissues.^38^ Proinflammatory Ccl12 has been suggested as a target for treating intestinal inflammation in mice with experimental colitis,^39^ while IL-22 is highly upregulated in inflamed intestinal mucosa and sera from patients with IBD.^40^

In this study, we also found KD could reduce the ILC3 cell production in the inflamed colonic epithelium and FMT indicated this effect was associated with KD-modified gut microbiota. ILC3s, developing from common lymphoid progenitor cells, have recently been proved to participate in not only maintaining gastrointestinal mucosal homeostasis but also contributing to IBD progression.^41^ The previous mechanism investigation revealed that ILC3 cells promote IL-17a, IL-22, and IL-18 production to aggravate IBD development through damaging intestinal mucosa barrier, which directly caters to our array results showing their levels were reduced in intestinal tissues of KD-treated mice.^12^ In addition, a newly published study has suggested microbial composition serves as a crucial factor affecting ILC3 cells during infectious or inflammatory diseases.^42^ Therefore, we speculated KD-modified gut microbiota exerted its anti-inflammatory effect partly through reducing ILC3 cell production. Future investigations are needed to clarify which KD-related bacteria species were dominantly responsible for ILC3 cell production in the inflammatory microenvironment.

There are some limitations to this study. Firstly, we found KD alleviated the progression of intestinal inflammation but meanwhile reduced the abundance of some healthy bacteria such as Lactobacillus as compared with ND. On the one hand, we explain this finding to the fact that intestinal inflammation is affected by the global changes of gut microbiota rather than one or several bacteria. On the other hand, future work is still needed to confirm the dominant bacteria responsible for the beneficial role of KD based on more accurate detecting techniques such as metagenomics. Secondly, we observed KD could also induce liver lipid accumulation in colitis mice, which is in accordance with a recent study showing similar effects in type 2 diabetic mice.^43^ This finding implies a potential health risk of long-term KD but another recent study has advocated high-intensity interval training (HIIT) may be helpful in preventing KD-induced adverse events.^44^ Therefore, whether HIIT could mitigate the liver lipid accumulation in KD-treated colitis mice will be investigated in our following work. Thirdly, we noted bacterial invasion of epithelial cells was over-represented in biological functions of gut microbiota from KD group, suggesting the interaction of KD-dominated bacteria with epithelial cells. In future, we will focus on some selected KD-dominated bacteria and try to clarify the impact of their interaction with epithelial cells on intestinal homeostasis and intestinal diseases. Lastly, our study was entirely based on murine models and the actual role of KD on human beings especially for those with or at risk of colitis remains unknown. This limitation is expected to be solved by well-designed clinical trials with ethical approve in future.

In conclusion, this study demonstrates for the first time that FMT from donors with a KD confers microbiota benefits and relieves colitis in DSS-induced recipients. We found that diets play an important role in shaping gut microbiota and that a KD can produce ideal gut microbiota for promoting weight control and resisting inflammation. Although this study is based on murine models and clinical effects have not yet been verified in human trials, we believe that our results provide new avenues for dietary IBD therapies and inform the customization and design of bacterial consortia for microbiota-targeted dietary supplements in patients with IBD to prevent intestinal barrier deterioration.

## Materials and methods

The study design is illustrated in Fig. 1a and consisted of two parts. Firstly, thirty-four 10-week-old male C57BL/6J mice were housed under specific pathogen-free conditions in Shanghai SLAC Laboratory Animal Co. Ltd, Shanghai, China, and were divided into three groups: KD (*n* = 10, KD contains 10% kcal protein, <1% kcal carbohydrate, and 89% kcal fat, FBSH Biotechnology Co. Ltd, Shanghai, China), LCD (*n* = 10, LCD contains 20% kcal protein, 10% kcal carbohydrate, and 70% kcal fat, FBSH Biotechnology Co. Ltd), and ND (*n* = 14, 18% kcal protein, 65% kcal carbohydrate, and 17% kcal fat). Mice were co-housed prior to starting the test diets to control for cage effects.^45^ Food intake was set at 11.9 kcal/day and decreased to 11.2 kcal/day after weight gain was observed during the first weeks of the study. Cross-sectional mice were always maintained on 11.2 kcal/day. A detailed description of diet composition is provided in Supplementary Table 4. After 16 weeks of dietary intervention, the mice were treated with or without 3% DSS for 1.5 weeks and defined as KD colitis (KD-C, *n* = 10), LCD colitis (LCD-C, *n* = 10), ND colitis (ND-C, *n* = 10), and ND (*n* = 4) groups.

Secondly, 34 germ-free C57BL/6J mice were bred at the Shanghai SLAC Laboratory Animal Co. Ltd, China. GF mice received FMT using feces from the dietary-treated mice and were treated with a normal diet for 2 weeks before being administered with 3% DSS for 1 week (normal diet) and divided into four groups: KD + FMT colitis (FKD + C, *n* = 10), LCD + FMT colitis (FLCD + C, *n* = 10), ND + FMT colitis (FND + C, *n* = 10), and ND + FMT (FND, *n* = 4). Body weight was measured weekly, while fecal and blood samples were collected from each cage before and after DSS treatment. The blood collection was performed using cardiac puncture. Epididymal white adipose, MLNs, liver tissue, and colon tissue were collected after sacrifice. Body mass index was calculated using Lee’s index [body weight (g) × 1000/body length (cm)]^1/3^. Colitis was assessed using the DAI and calculated based on weight loss percentage, diarrhea, and haematochezia. All procedures were carried out according to protocols approved by the Animal Care and Use Committee of Shanghai Tenth People’s Hospital affiliated to Tongji University.

See Supplementary Materials for details on fecal transplantation, serum metabolic measurement, 16S rDNA microbiota profiling, bioinformatics analysis, metabolomics analysis by gas chromatography–mass spectrometry (GC-MS), metabolomics analysis by ultra-high-performance liquid chromatography-high resolution mass spectrometry (UHPLC-HRMS/MS), metabolomics data analysis, hematoxylin and eosin (H&E) staining and histopathological evaluation, measurement of hepatocyte fat deposition, immunofluorescence staining, RNA extraction and quantitative RT-PCR, RT2 profiler PCR array gene expression, transcriptome sequencing, and statistical analysis.

## Supplementary information

Supplementary data

Supplementary Table 1

Supplementary Table 2

Supplementary Table 3

## Data Availability

All the 16S sequencing data were submitted to the National Center for Biotechnology Information Sequence Read Archive (accession number SRP299843). Additional data related to this paper may be requested from the authors. Email: yanleima@fudan.edu.cn (Y.M)
