# Supplementary material for: Ketogenic diet alleviates colitis by reduction of colonic group 3 innate lymphoid cells through altering gut microbiome
Source: Signal Transduct Target Ther. 2021 Apr 23;6:154. doi: 10.1038/s41392-021-00549-9 (PMC8062677; doi:10.1038/s41392-021-00549-9)
Supplement: Supplementary file 1 — Supplementary data [file 41392_2021_549_MOESM1_ESM.pdf]

Supplemental Information

Ketogenic diet alleviates colitis by reduction of colonic group 3 innate lymphoid cells through altering gut microbiome

Cheng Kong, Xuebing Yan, Yongqiang Liu, Linsheng Huang, Yefei Zhu, Jide He, Renyuan Gao, Matthew F. Kalady, Ajay Goel, Huanlong Qin, and Yanlei Ma

Correspondence to: Yanlei Ma (yanleima@fudan.edu.cn), Huanlong Qin, (hl-qin@hotmail.com), and Ajay Goel, (ajgoel@coh.org).

This PDF file includes:

Supplementary Materials
Figures S1 to S3
Tables S4 to S5

Other Supplementary Materials for this manuscript include the following:

Table S1 (Excel document)
Table S2 (Excel document)
Table S3 (Excel document)

Supplementary Materials

16S rDNA microbiota profiling

Total DNA was extracted from fecal samples as described previously [1]. 16S rDNA high-throughput sequencing was performed using an Illumina HiSeq PE250 (Illumina, San Diego, CA, USA). Variable regions V3–V4 on bacterial 16S rDNA genes were amplified using forward (F341 5'-ACTCCTACGGGRSGCAGCAG-3') and reverse (R806 5'-GGACTACVVGGGTATCTAATC-3') primers. Raw data were subjected to quality control using UPARSE. Qualified reads were clustered to generate operational taxonomic units (OTUs) at 97 % similarity using Usearch [2].

Bioinformatics analysis

Alpha diversity was used to assess the complexity of species diversity for each sample via three indices (Chao1, Shannon, and Simpson) in QIIME software. Principle coordinates analysis (PCoA), heatmap analysis, Bray-Curtis similarity clustering, and species abundance analysis were performed using R version 3.3.0. The linear discriminant analysis effect size method (http://huttenhower.sph.harvard.edu/lefse/) was used to analyze microbial features.

Metabolomics analysis by gas chromatography–mass spectrometry (GC-MS)

Briefly, frozen samples (40 mg) were firstly treated with pyridine hydrochloride solution. Sample preparation was carried out as described previously [3]. Samples were analyzed using an Agilent 7890A/5975C GC-MS system (Agilent Technologies Inc., Palo Alto, CA, USA), with an OPTIMA® 5 MS Accent fused-silica capillary column (Machery-Nagel, Düren, Germany) to separate the derivatives. Helium (> 99.999 %)

was used as a carrier gas at a constant flow rate of 1 mL/min, injection volume of 1 μL, and solvent delay time of 5.4 min. The initial oven temperature was held at 60 °C for 1 min, ramped to 240 °C at a rate of 12 °C/min, to 320 °C at 40 °C/min, and held at 320 °C for 4 min. The electron ionization energy was 70 eV. Data were collected in full scan mode (m/z 50-600).

Metabolomics analysis by ultra-high-performance liquid chromatography-high resolution mass spectrometry (UHPLC-HRMS/MS)

Chromatographic separation was performed using a ThermoFisher Ultimate 3000 UHPLC system (ThermoFisher Scientific, SantaClara, CA, USA) with a Waters HSS T3 column (Waters, Milford, MA, USA) at a column temperature of 40 °C, flow rate of 0.4 mL/min, and injection volume of 1 μL. Linear gradient elution was performed with the following program: 0 min, 1 % B held to 2 min; 11 min, 100 % B held to 14 min; 14 min, 1 % B and held to 16 min. Eluents were analyzed on a ThermoFisher Q Exactive™ Hybrid Quadrupole-Orbitrap™ mass spectrometer (ThermoFisher Scientific) in heated electrospray ionization (HESI) positive (+) and negative (-) modes with a spray voltage of 4000 V. Capillary and probe temperatures were 320 and 350 °C for HESI+ mode or 250 and 300 °C for HESI- mode, respectively. Full scans were carried out at a high resolution of 70000 full width at half maxima (m/z 200) with a range of 70-1000 m/z and an AGC target of 1×10^6.

Metabolomics data analysis

For multivariate statistical analysis, normalized data were imported into soft independent modeling of class analogy (SIMCA) software (version 14.1, AB Umetrics,

Umeå, Sweden) and preprocessed by UV scaling and mean centering before principal components analysis (PCA), partial least squares discrimination analysis (PLS-DA), and orthogonal PLS-DA (OPLS-DA) were performed. Model quality was described by R^2X or R^2Y and Q^2. R^2X (PCA) or R^2Y (PLS-DA and OPLS-DA) are defined as the proportion of variance in the data explained by the models and indicates goodness of fit. Q^2 is defined as the proportion of variance in the data predicted by the model and indicates the predictability of the current model as calculated by cross-validation. To avoid over-fitting, default seven-round cross-validation was performed in SIMCA to determine the optimal number of principal components. For univariate statistical analysis, normalized data were analyzed using the "muma" package in R (version 3.5.1), in which parametric tests were performed on normally distributed data using Welch's *t* test and nonparametric tests were performed on non-normally distributed data using the Wilcoxon Mann-Whitney test. Variable importance in the projection (VIP) values for the OPLS-DA model > 1 and p values from univariate statistical analysis < 0.05 were identified as potential differential metabolites.

Transcriptome sequencing

Total RNA was isolated and purified using TRIzol reagent (Invitrogen) following the manufacturer's procedure. The RNA amount and purity of each sample was quantified using NanoDrop ND-1000 (NanoDrop, Wilmington, DE, USA). The RNA integrity was determined using Bioanalyzer 2100 (Agilent, CA, USA), and confirmed by electrophoresis with denaturing agarose gel. Poly (A) RNA is purified from 1μg total RNA using Dynabeads Oligo (dT)25-61005 (Thermo Fisher, CA, USA). Then the

poly(A) RNA was fragmented into small pieces using Magnesium RNA Fragmentation Module (NEB, cat.e6150, USA). After that, the cleaved RNA fragments were reverse-transcribed into cDNA by SuperScript™ II Reverse Transcriptase (Invitrogen, cat. 1896649, USA). Finally, the sequence analysis was performed on an Illumina Novaseq™ 6000 following the manufacturer's instructions. After generating the final transcriptome, the expression levels of all transcripts were estimated. The differentially expressed mRNAs were selected using the following criterion: fold change > 2 or fold change < 0.5 and p value < 0.05.

Hematoxylin and eosin (H&E) and immunohistochemistry staining and histopathological evaluation

Paraffin-embedded colon tissues were cut into 5-μm sections, dewaxed in xylene for 10 min, and dehydrated in gradient alcohol. The sections were then stained with H&E, PAS Stain Kit (Abcam, Cambridge, MA, USA), and anti-MUC2 antibodies (1:2000, Abcam) according to standard protocols, dehydrated, and then sealed and examined under a light microscope (Nikon Eclipse 80i, Tokyo, Japan). Histopathological evaluation was performed by two researchers blinded to the details of each section using the following evaluation criteria: 0, no inflammation; 1, moderate inflammation in the basal layer; 2, moderate mucosal hyperplasia or severe inflammation; 3, severe mucosal hyperplasia; 4, crypt absence or ulceration.

Immunofluorescence staining

Sections were soaked in dimethyl benzene for 10 min, followed by 100, 95, and 70 % alcohol treatment for 5 min. Next, 2000 mL of 0.01 mol/L natrium citricum buffer

solution (pH 6.0) was added to a pressure kettle in which the colon sections were placed on a staining rack. Goat serum was added to the samples for 20 min at room temperature and the supernatant was removed. The samples were then incubated with primary monoclonal antibodies diluted at 1:50 (occludin, ZO-1) in blocking buffer overnight at 4 °C, incubated at 37 °C for 45 min, and washed with PBS for three times. Samples were incubated with secondary antibodies diluted at 1:100 (FITC-conjugated anti-rabbit; Abcam) in blocking buffer for 30 min at room temperature. Next, samples were washed with PBS for three times, incubated with 5-10 mL of DAPI staining solution for 10 min, and washed with PBS for four times for 5 min. One drop of 50 % glycerine was added to each section, and laser confocal microscopy (Nikon, Japan) was used to detect occludin and ZO-1 expression. RORγt (Millipore, billerica, MA, USA) and CD3 (Novus, Littleton, CO, USA) antibodies were used to detect RORγt^{+}CD3^{-} ILC3 cells as described previously [4].

References

1 Kong, C. *et al.* Probiotics improve gut microbiota dysbiosis in obese mice fed a high-fat or high-sucrose diet. *Nutrition*. **60**, 175-184, (2018).

2 Edgar, R. C. UPARSE: highly accurate OTU sequences from microbial amplicon reads. *Nature methods*. **10**, 996-998, (2013).

3 Liu, Y. *et al.* The Association of Post-Stroke Cognitive Impairment and Gut Microbiota and its Corresponding Metabolites. *J Alzheimers Dis*. **73**, 1455-1466, (2020).

4 Kim, S. *et al.* CD117^{+} CD3^{-} CD56^{-} OX40Lhigh cells express IL-22 and display an LTi phenotype in human secondary lymphoid tissues. *Eur J Immunol*. **41**, 1563-1572, (2011).

Fig. S1

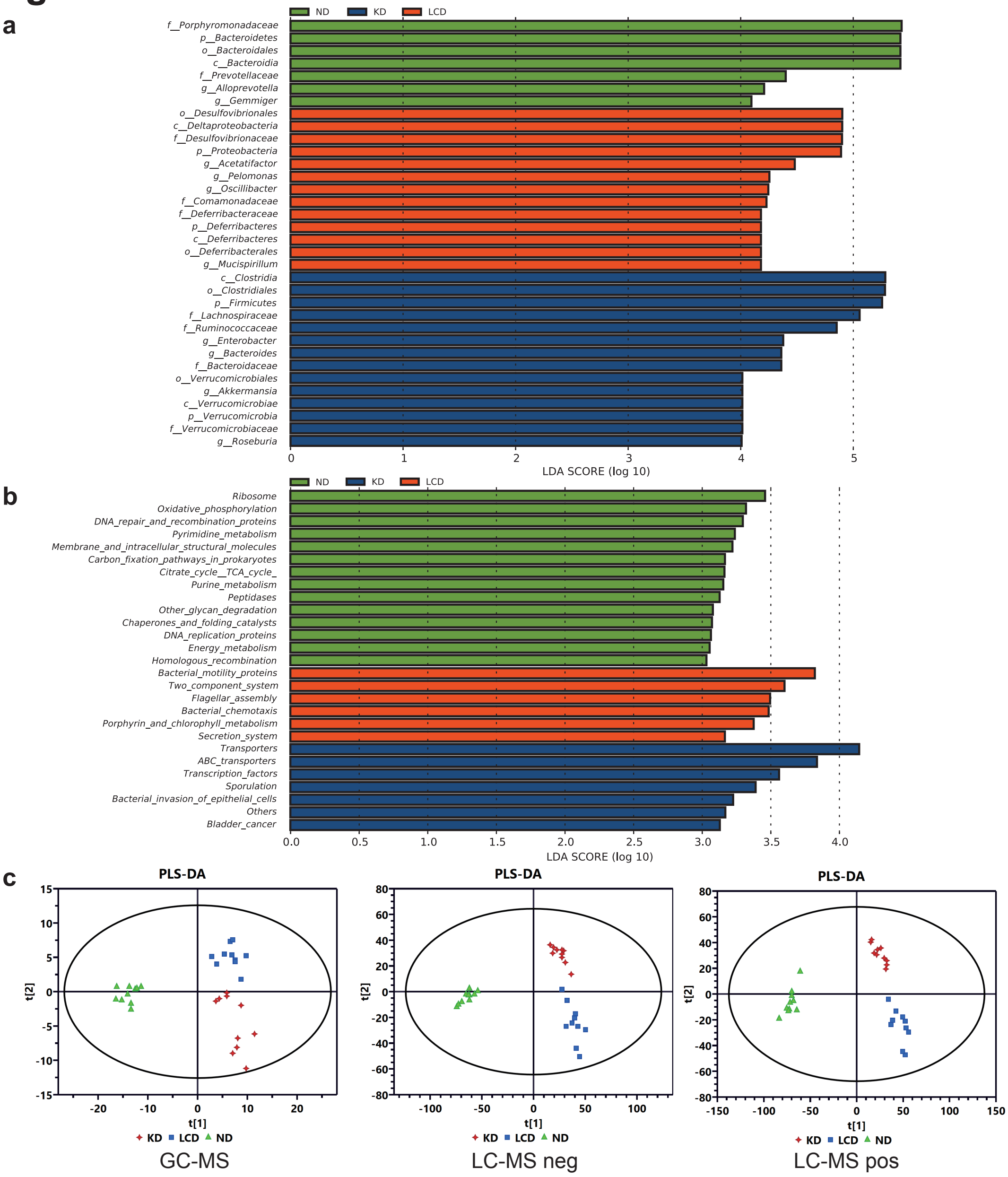

Supplementary Fig. 1 Effect of KD and LCD on gut microbiota and metabolism in mice before colitis induction.

a Histogram of linear discriminant analysis scores for differentially abundant bacteria in mice fed with a KD, LCD, or ND for 16 wk (p: Phylum, c: Class, o: Order, f: Family, g: Genus). **b** Differentially abundant KEGG pathways in each group were identified by LEfSe. LDA score > 3.0, $p < 0.05$. **c** PLS-DA score plots derived from ultra-high-performance liquid chromatography-high resolution mass spectrometry (UHPLC-HRMS/MS) electrospray ionization (ESI) (+), UHPLC-HRMS/MS ESI (−), and gas chromatography–mass spectrometry (GC-MS) spectra in each group. KD, ketogenic diet; LCD, low-carbohydrate diet; ND, normal diet; LEfSe, linear discriminant analysis effect size; PLS-DA, partial least squares discrimination analysis.

Fig. S2

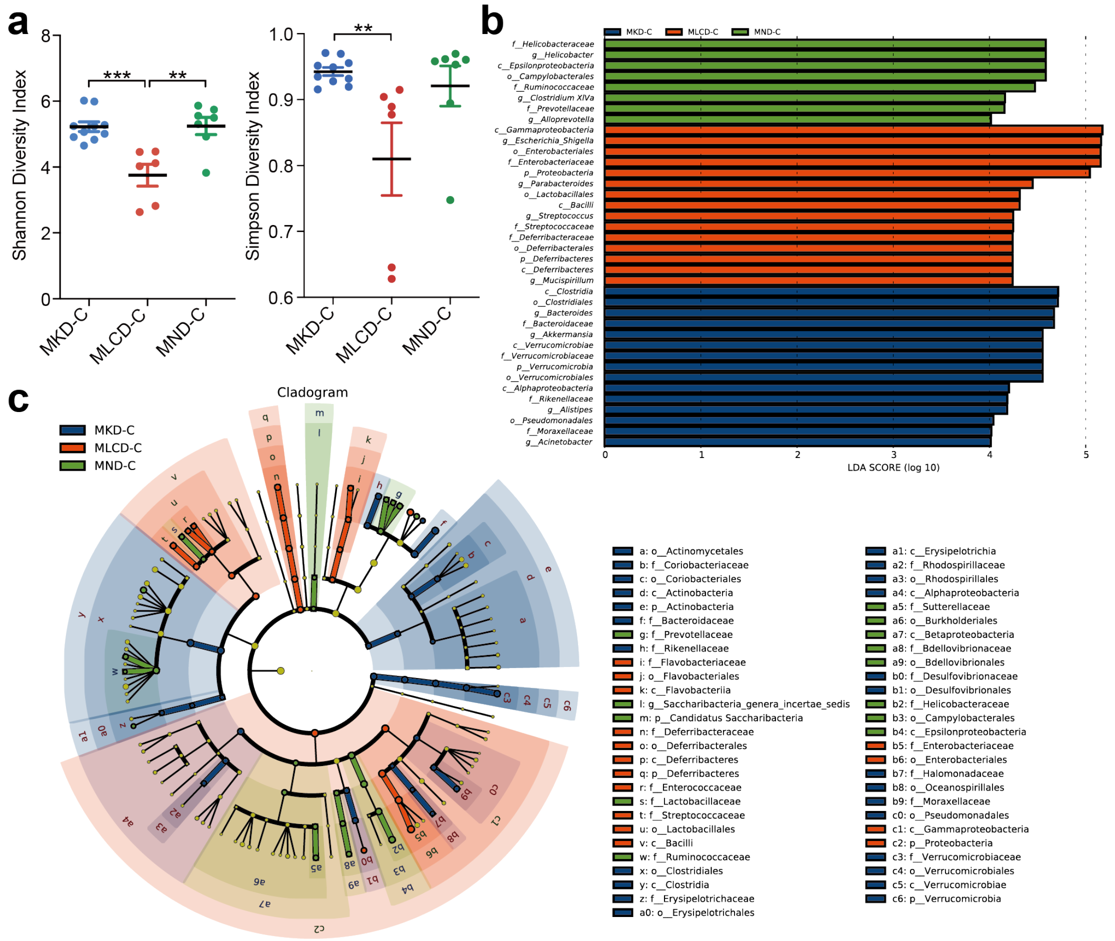

Supplementary Fig. 2 Effects of KD and LCD on the MLN microbiome of mice with colitis.

a α-diversity Simpson and Shannon indices in the MLN microbiome of mice with DSS-induced colitis fed with KD (MKD-C), LCD (MLCD-C), or ND (MND-C). **b-c** Histogram (**b**) and cladogram (**c**) of linear discriminant analysis scores for differentially abundant bacteria (p: Phylum, c: Class, o: Order, f: Family). $^{**}p < 0.01$; $^{***}p < 0.001$. Results are expressed as mean ± SEM (**a**). KD, ketogenic diet; LCD, low-carbohydrate diet; ND, normal diet; MLN, mesenteric lymph node.

Fig. S3

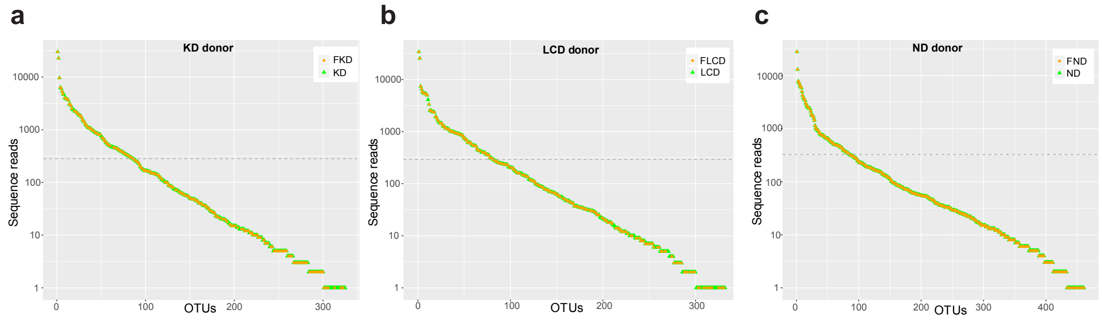

Supplementary Fig. 3 Validation for the efficacy of fecal microbiota transplantation in germ-free mice.

a-c Total read counts of OTUs present in KD (**a**), LCD (**b**), ND (**c**) donor fecal microbiota (green triangles). Orange dots indicate OTUs present in recipient mice after 14-day fecal microbiota transplantation. Horizontal dashed line separates the top 80 most abundant OTUs. KD, ketogenic diet; LCD, low-carbohydrate diet; ND, normal diet; DSS, dextran sulfate sodium; OTU, operational taxonomic units.

Table S4. Detailed description of dietary composition.

Ingredient (g/kg diet)	Control	Low-carbohydrate	Ketogenic
Protein, of which	203	316.7	183.7
Casein	*200*	*312*	*181*
L-cystein	*3*	*4.7*	-
D-methionine	-	-	*2.7*
Carbohydrates, of which	630	112	-
Corn starch	*398*	-	-
Maltodextrin	*132*	-	-
Sucrose	*100*	*112*	-
Fat, of which	70	423	631
Soybean oil	*70*	*70*	*70*
Lard	-	*353*	*561*
Choline bitartrate	2.5	2.5	‡
Cellulose (73.5 mg/day)	50	71	85
Tert-butylhydroquinone	0.014	0.085	0.126
Mineral mix	35	60	60
Vitamin mix	10	15	13
Other minerals	-	-	27.5

‡, included in the vitamin mix

Table S5. Primer Sequences for Real-time polymerase chain reaction.

Gene	Sequence 5'-3'
IL-17α	Forward primer: GAAGGCCCTCAGACTACCTC<br>Reverse primer: CAGCATCTTCTCGACCCTGA
IL-22	Forward primer: GCCAGCCTTGCAGATAACAA<br>Reverse primer: GTTTGGTCAGGAAAGGCACC
IL-18	Forward primer: TGGATCCATTTCCTCAAAGG<br>Reverse primer: TGGATCCATTTCCTCAAAGG
CCL4	Forward primer: CTCCCACTTCCTGCTGTTTCT<br>Reverse primer: GTTCAACTCCAAGTCACTCATGT
ZO1	Forward primer: CCAGCAACTTTCAGACCACC<br>Reverse primer: TTGTGTACGGCTTTGGTGTG
Occludin	Forward primer: TAAGAGCTTACAGGCAGAACTAG<br>Reverse primer: CTGTCATAATCTCCCACCATC
GAPDH	Forward primer: ATGGGTGTGAACCACGAGA<br>Reverse primer: CAGGGATGATGTTCTGGGCA
